# Artificial Intelligence in Clinical Decision-Making: A Scoping Review of Rule-Based Systems and Their Applications in Medicine

**DOI:** 10.7759/cureus.91333

**Published:** 2025-08-31

**Authors:** Ashraf Alnattah, Mahdie Jajroudi, Seyyed Ali N Fadafen, Mahdi N Manzari, Saeid Eslami

**Affiliations:** 1 Department of Medical Informatics, School of Medicine, Mashhad University of Medical Sciences, Mashhad, IRN; 2 Pharmaceutical Research Center, Mashhad University of Medical Sciences, Mashhad, IRN; 3 Department of Medical Informatics, University of Amsterdam, Amsterdam, NLD

**Keywords:** clinical, drug, engine, medicine, rule

## Abstract

Artificial intelligence (AI) has become increasingly integrated into clinical workflows, with rule-based clinical decision support systems (CDSS) emerging as one of its most mature and widely adopted applications. These systems rely on rule engines, that is, software components that apply predefined conditional logic (if/then rules) to patient data, to deliver alerts, diagnostic suggestions, or treatment recommendations. By embedding expert knowledge into structured rule sets and utilizing inference engines to process them, rule-based CDSS provides transparent, interpretable, and adaptable decision support. Although their use has expanded significantly over the past decade, evolving from simple decision aids to advanced tools incorporating AI and real-time analytics, a comprehensive synthesis of their applications, effectiveness, and technological evolution remains lacking. This scoping review aims to examine the current landscape of rule engine implementations in medicine, focusing on their clinical functionalities, evaluated outcomes, technological characteristics, and geographic adoption patterns across different medical domains. Following established scoping review methodology, we conducted a systematic search of PubMed and Scopus databases (2007-2023). Of 437 initially identified records, 28 studies met our inclusion criteria after rigorous screening. Data were extracted on study characteristics, clinical applications, rule engine technologies, and implementation outcomes, with particular attention to temporal trends and geographic distribution. The analysis revealed several key findings. The United States accounted for 46.42% of studies, demonstrating significant geographic concentration. Technologically, implementations evolved from early SQL-based systems to contemporary approaches integrating machine learning and natural language processing. Clinically, the rule engine showed particular effectiveness in chronic disease management (approximately 30% focused on diabetes care) and demonstrated measurable improvements, such as 30% reductions in adverse drug events. However, challenges persisted in system interoperability and clinician adoption across multiple studies. Our analysis of 28 studies demonstrates that rule engines have demonstrated substantial potential to enhance clinical decision-making and healthcare efficiency, though their adoption remains uneven geographically and is technically constrained in some settings.

Based on our findings, we recommend: (1) developing standardized implementation frameworks to address interoperability challenges, (2) expanding research and deployment in underrepresented regions, and (3) investing in hybrid systems that combine rule-based logic with machine learning capabilities. These insights provide valuable guidance for healthcare organizations seeking to implement or optimize rule engine technologies in clinical practice.

## Introduction and background

Over the past decade, the medical community has made significant efforts to harness the potential of artificial intelligence (AI) and digital technology, aiming to enhance medical practices and adapt to the evolving technological landscape. Among the various AI applications, the design of decision support systems using the rule engine has emerged as a critical approach to optimize healthcare delivery and reduce medication errors [1]. Some studies have demonstrated the ability of rule engine technology to mitigate errors and improve the quality of medical care [2].

A rule engine system, which is one of the important parts of AI, is known for its ability to adapt to ever-changing situations. These systems consist of a set of rules (usually decomposed into rule sets) and an inference engine that processes these rules [3]. On the other hand, a rule engine refers to software that executes one or more rules within a runtime production environment, with each rule engine employing its proprietary rule-storage formats and varying features.

The marketplace offers numerous rule engines that support a variety of use cases across industries. These include both commercial and open-source systems developed by organizations and individuals worldwide. For reference, Table 1 lists the rule engines mentioned in this article along with their respective manufacturers, cities, and countries of origin. All product names are trademarks or registered trademarks of their respective holders.

**Table 1:** List of rule engines and their manufacturers/developers

Sr. No.	Software name	Manufacturer/developer	City	Country
1	Drools (JBoss© Rules)	Red Hat	Raleigh	USA
2	SEBASTIAN©	(Manufacturer details not publicly available)	–	–
3	Jena™	Apache Software Foundation	Forest Hill	USA
4	OpenCDS©	OpenCDS Community (University of Utah)	Salt Lake City	USA
5	iLog®	IBM	Armonk	USA
6	SQL	IBM (originally; now supported by multiple vendors)	Armonk	USA
7	Prolog®	Marseille-Prolog (early implementation)	Marseille	France
8	OPS5	Carnegie Mellon University	Pittsburgh	USA
9	JRules®	IBM	Armonk	USA
10	Blaze Advisor®	Fair Isaac Corporation (FICO)	San Jose	USA
11	Mandarax	Lars Heuer	Bremen	Germany
12	ECA	Not a product; implemented by various vendors	–	–
13	JLisa	(Manufacturer details not publicly available)	–	–
14	Take	(Manufacturer details not publicly available)	–	–
15	Jess®	Sandia National Laboratories	Albuquerque	USA
16	CLIPS®	NASA	Houston	USA
17	OpenRules®	OpenRules, Inc.	Mountainside	USA
18	JXBRE®	Arnaud Bailly	Paris	France
19	JEOPS®	University of São Paulo	São Paulo	Brazil
20	Roolie®	(Manufacturer details not publicly available)	–	–
21	Termware	SoftElegance	Kyiv	Ukraine
22	JRuleEngine	(Manufacturer details not publicly available)	–	–
23	Zilonis®	Zilonis, Inc.	Munich	Germany
24	DTRules®	David C. Proctor	Westford	USA

Despite the development and widespread implementation of rule engines, there remains a dearth of published studies, specifically dedicated to this branch of artificial intelligence. Historically, Nelson and Sen [4] observed the emergence of the modern business rules (BRs) segment as distinct from the AI and expert systems arenas during the mid to late 1980s. In 1987, Beau Sheil introduced the concept under the names "Rule interpreters" and "Rule-based programming" as fragments of AI software technology [5]. Thus, the term "rule engine" finds its roots in "Business rule engine".

As documented by Chisholm (2004) and Morgan (2002), the GUIDE (an IBM-oriented industry user group, now defunct) originally defined a business rule as “a statement that defines or constrains some aspect of the business” [6,7]. It is intended to assert business structure or to control or influence the behavior of the business.

Today, rule engines find applications in diverse domains such as finance, healthcare, retail, manufacturing, and more [8]. In essence, rule engine systems are technical software systems that apply conditional actions (if/then rules) to data.

One of the distinguishing features of rule engine systems is their remarkable flexibility and adaptability to dynamically changing working environments and guideline updates.

In the medical field, numerous daily tasks, decisions, and routines necessitate the utilization of these modern technologies, aligning with the demands of the current era.

A rule engine can be used as a clinical decision-support tool in medicine in several ways: rule engines can automate decision-making and implement business logic to enforce policies and protocols in healthcare settings [9]; they can support real-time decision-making for patient monitoring, diagnostics, treatment, and medication management [10]; recent research has focused on using rule engines to control the flow of clinical guidelines and invoke rule execution to interface with local applications.

For example, a rule engine could be used to automatically check a patient's medical history, current medications, and lab results against a set of predefined rules [11]. Based on this analysis, the rule engine could then provide recommendations to the clinician on the most appropriate course of treatment [12]. This can help ensure consistent, evidence-based care and reduce the risk of medical errors.

Overall, rule engines offer a flexible and scalable way to incorporate clinical decision support into healthcare IT systems, helping to improve patient outcomes and the quality of care [13].

This scoping review aims to elucidate the role of rule engines and their applications in medical research, providing a panoramic view of the current state of affairs. To the best of our knowledge, this scoping review represents the first comprehensive literature review in this field.

This scoping review was previously posted to the Research Square preprint server on January 12, 2024.

Research questions

This scoping review was guided by the following research questions (Table 2), designed to comprehensively examine the implementation and impact of rule engines in healthcare.

**Table 2:** Research questions guiding the scoping review SQL: Structured Query Language; NLP: Natural Language Processing

S. No.	Category	Research question
1	Functionalities and applications	What specific clinical and administrative tasks have rule engines been used to accomplish across different medical domains?
2	Evaluated outcomes	What measurable clinical outcomes (e.g., error reduction) and operational outcomes (e.g., workflow efficiency) have been demonstrated in peer-reviewed studies?
3	Technological implementations	Which rule engine technologies (e.g., Drools, SQL-based) and complementary tools (e.g., NLP) are most prevalent in recent implementations?
4	Technological implementations	How have the technical approaches evolved over time in terms of complexity and integration capabilities?
5	Implementation challenges	What are the most frequently reported barriers to successful implementation (e.g., interoperability issues, clinician adoption)?
6	Implementation challenges	What strategies have proven effective for overcoming these challenges in different healthcare environments?

## Review

Methods

This scoping review was conducted following the methodological framework proposed by Arksey and O’Malley [14], and it adheres to the PRISMA-ScR (Preferred Reporting Items for Systematic Reviews and Meta-Analyses extension for Scoping Reviews) guidelines [15]. The methodology was executed in five key stages: (1) identifying the research questions; (2) searching for relevant studies; (3) selecting eligible studies; (4) charting the data; and (5) collating, summarizing, and reporting the results. Three independent reviewers (AA, MJ, and AN) participated in the screening and data extraction process. Any discrepancies during the selection or data abstraction phases were resolved through discussion and consensus.

Identification of Relevant Studies

A comprehensive literature search was conducted across PubMed and Scopus databases to identify relevant studies published between 2007 and 2024. The search strategy employed controlled vocabulary and keywords associated with rule engine implementation in medical and clinical contexts (Table 3).

**Table 3:** Search strategies and key terms used for identifying relevant studies in PubMed and Scopus databases (2007–2023)

Database	Search terms
Scopus	TITLE-ABS-KEY ( "rule engine" AND ( "medica?" OR " drug" OR "clinical" ) ) AND ( LIMIT-TO ( DOCTYPE , "ar" ) OR LIMIT-TO ( DOCTYPE , "cr" ) OR LIMIT-TO ( DOCTYPE , "re" ) ) AND ( LIMIT-TO ( SUBJAREA , "MEDI" ) OR LIMIT-TO ( SUBJAREA , "HEAL" ) ) AND ( LIMIT-TO ( EXACTKEYWORD , "Humans" ) OR LIMIT-TO ( EXACTKEYWORD , "Rule Engine" ) OR LIMIT-TO ( EXACTKEYWORD, "Decision Support Systems")) AND ( LIMIT-TO (LANGUAGE, "English")) AND ( LIMIT-TO ( SRCTYPE, "j"))
PubMed	"rule engine" Filters applied: Clinical Trial, Meta-Analysis, Randomized Controlled Trial, Review, Systematic Review, From 2007/1/1 to 2023/12/31, Humans, English

To ensure a comprehensive and focused review, studies were included if they specifically addressed the application or implementation of rule engines within clinical or healthcare settings, were published in peer-reviewed journals, written in English, and fell within the publication timeframe of 2007 to 2023. The 2007-2023 timeframe was selected to capture the complete evolution of modern rule-engine implementations while ensuring all included publications had undergone full peer review cycles by our search date (January 2024). Studies were excluded if they originated from non-medical fields (such as engineering or computer science without a direct clinical application) or if they did not clearly focus on rule engine technologies. In addition, non-original works, including reviews, books, conference abstracts, and articles lacking peer review, were also excluded. A detailed overview of the inclusion and exclusion criteria is provided in Table 4.

**Table 4:** Inclusion and exclusion criteria applied for study selection in this scoping review of rule engine applications in medicine CDSS: clinical decision support systems

Criterion	Inclusion	Exclusion
Study focus	Studies where “rule engine” is utilized	Studies used CDSS alone without “rule engine”
Study discipline	Medicine	Any engineering, computer science, and non-medical specialty
Type of articles	Original research published in a peer-reviewed journal	Exclude review articles, symposiums, books, series, trade journals, and undefined articles
Language	Language is English	Non-English languages
Time period	2007-2023	Studies outside these dates

Data Charting

To ensure consistency and depth in analysis, a structured data extraction process was carried out using a predefined framework. Key information was collected from each study, including publication year, country of origin, study design, clinical domain, and the specific rule engine technology applied, such as Drools, SQL-based systems, or vendor-specific platforms. The use of additional tools, including natural language processing and machine learning, was also documented when relevant. Implementation outcomes were recorded, covering both clinical improvements and operational enhancements. Any reported challenges or limitations, such as integration difficulties or user resistance, were carefully noted. All extracted data were organized in Microsoft Excel 2021 (Microsoft Corp., Redmond, WA) to support clear categorization and facilitate comparison across studies.

Data Collation and Summarization

The final stage involved synthesizing and analyzing the extracted data thematically. Studies were grouped and compared by (1) timeframe of publication (pre-2015, 2015-2020, post-2020); (2) geographic distribution; (3) clinical application domain; (4) rule engine technologies; and (5) implementation outcomes.

This structured synthesis allowed us to track trends in tool adoption (e.g., structured query language or SQL to machine learning-based systems), clinical impact across medical fields, and persistent challenges in rule engine integration and use.

Results

**Figure 1:**
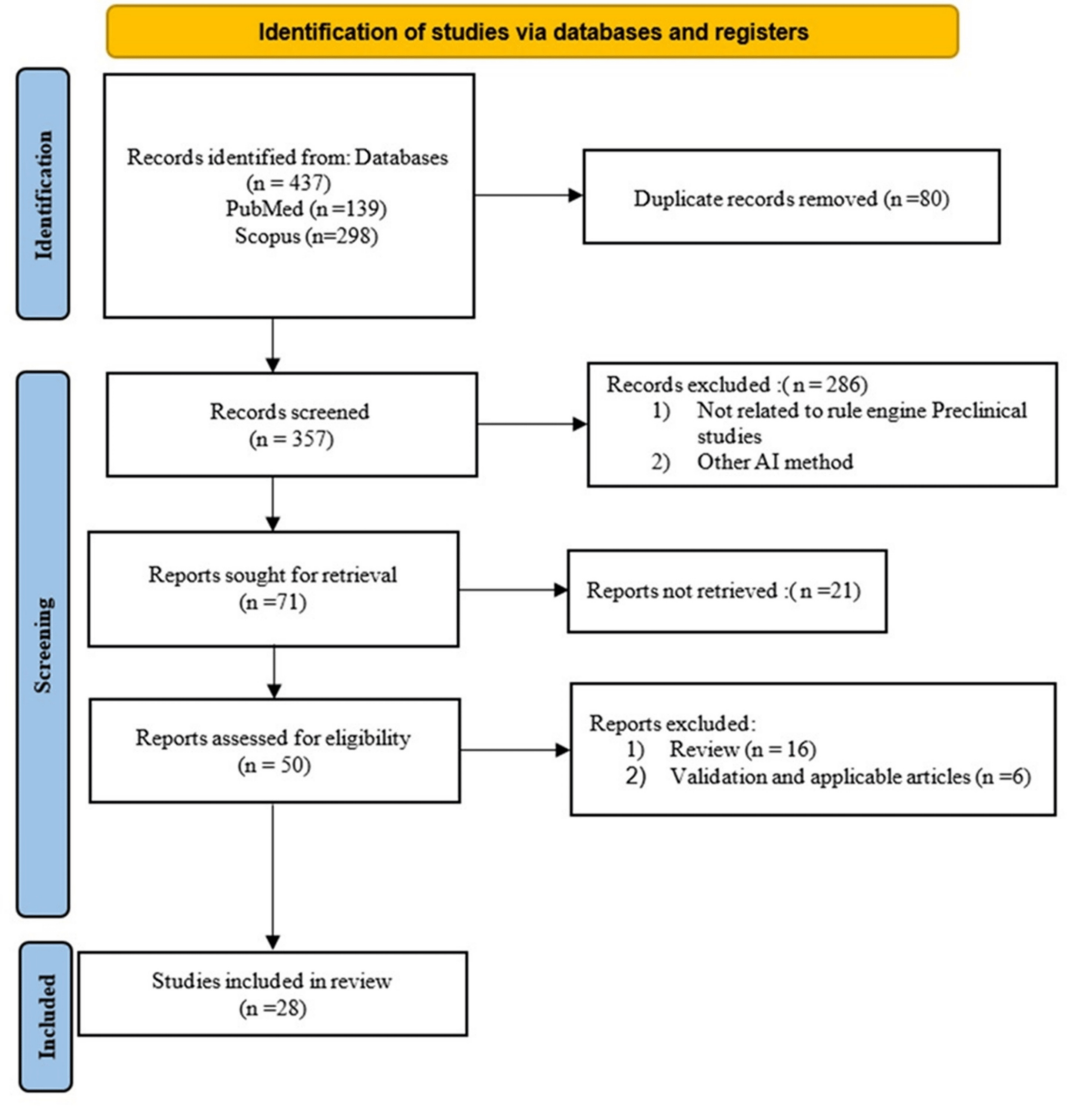
PRISMA-ScR flow diagram illustrating the selection process of included studies PRISMA-ScR: Preferred Reporting Items for Systematic Reviews and Meta-Analyses extension for Scoping Reviews

A comprehensive analysis of published literature was conducted to examine the application of rule engines in the medical domain. The initial search retrieved 437 records. After removing duplicates and conducting a multi-stage screening of titles, abstracts, and full texts, 28 studies met the inclusion criteria for this scoping review. The selection process is summarized in the PRISMA-ScR flow diagram (Figure 1).

**Figure 2:**
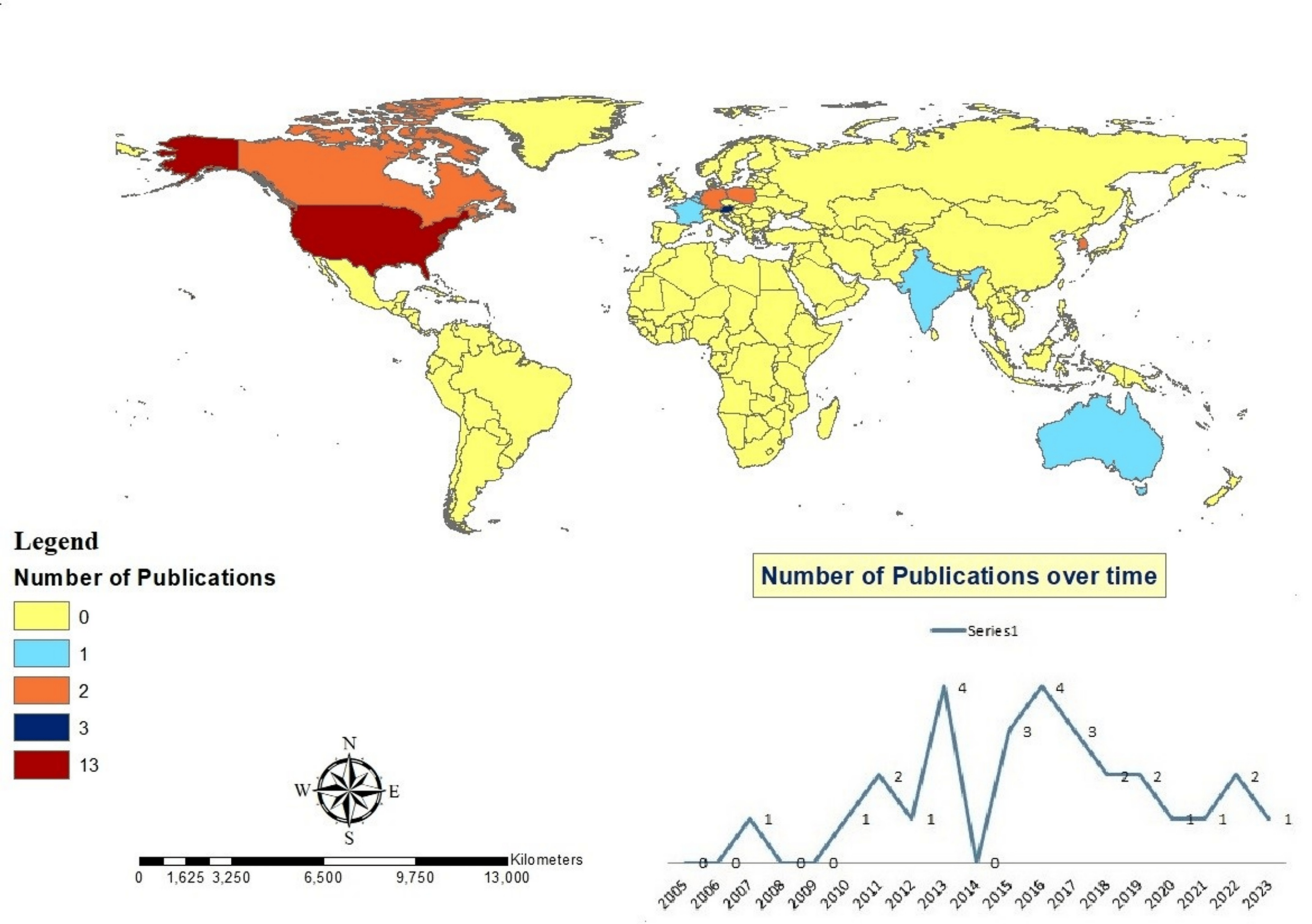
Geographic distribution and temporal trends of included studies on rule engine applications in healthcare This figure illustrates the number of included studies by country, highlighting regional contributions to the literature. It also depicts the progression of research activity over time, showing increased publication frequency in recent years, particularly after 2015, reflecting a growing interest in rule engine technologies within the medical domain. The figure was created by the authors using ArchGIS 10.8.

The included studies, published between 2007 and 2023, were authored by 185 researchers affiliated with institutions in 12 different countries. The United States was the most prominent contributor, accounting for 46.4% of the included articles (n = 13). Austria followed with 10.7% (n = 3), while South Korea, Poland, and Canada each contributed 7.1% (n = 2). The remaining studies were distributed among several other countries, each represented by a single article. This geographic pattern highlights a concentration of research activity in a few high-income countries, which may reflect disparities in access to funding, technical infrastructure, and institutional capacity to implement and evaluate rule engine technologies in healthcare (Figure 2).

Characteristics of Articles Included in this Scoping Review

The reviewed studies demonstrated regional and methodological diversity. In North America, research primarily focused on the detection and management of adverse drug events (ADEs), as well as strategies to improve hospital efficiency. In Europe, there was an emphasis on standardized clinical decision-support tools, including the use of Arden Syntax and decision tables aimed at optimizing clinical workflows and resource allocation. Studies conducted in Asia showcased innovative models such as remote healthcare systems and mobile platforms, particularly for managing chronic conditions like diabetes. From a temporal perspective, earlier studies published between 2007 and 2013 largely explored the feasibility and foundational performance of rule-based systems. In contrast, more recent research, especially from 2019 to 2023, has increasingly integrated artificial intelligence and advanced analytics to support real-time, personalized clinical decision-making (Table 5).

**Table 5:** Summary of rule engine applications across medical domains, highlighting tools, article counts, implementation timeframes, and representative examples SQL: Structured Query Language; BPM: business process management; NLP: Natural Language Processing

Application in medical field	Tools/methods used	Number of articles	Timeframe	Examples of use
Data management	SQL	5	Pre-2015	Managing structured patient data, identifying drug interactions.
Workflow optimization	BPM	3	Pre-2015	Hospital workflow management, resource allocation.
Clinical decision support	Drools, Arden syntax	4	2015-2020	Creating clinical alerts, implementing ICU scoring systems.
Text analysis	NLP	4	2015-2020	Extracting information from electronic health records, disease classification.
Predictive analytics	Supervised machine learning	1	Post-2020	Predicting patient outcomes, analyzing large datasets.
Real-time monitoring	Cache-based rule engines	1	Post-2020	Monitoring patient vitals, generating alerts for critical conditions.
Uncertainty management	Fuzzy Arden syntax	1	Post-2020	Handling uncertain medical data, aiding diagnosis in complex cases.
Vendor-based systems	Epic systems	1	Post-2020	Streamlining care processes, integrating rule engines with electronic systems.

Of the 28 included articles, 25 studies (89%) were original research published in peer-reviewed journals. In terms of research methodology, 13 studies (46%) employed a descriptive or formative design, while 4 studies (14%) used randomized controlled trials. Two studies (7%) applied data mining approaches, two (7%) focused on performance evaluation, and two (7%) addressed system implementation. One study (4%) used a pre-post comparison design based on contingency tables, and another (4%) adopted a retrospective method. Additionally, three studies (11%) did not clearly specify their research methodology (Table 6).

**Table 6:** Studies characteristics, objectives, and challenges REDCap: research electronic data capture; TAs: titration algorithms; NS: not specified; NLP: Natural Language Processing; HA-VTE: hospital-acquired venous thromboembolism; HAIs: healthcare-associated infections; EHR: electronic health record; CDS: clinical decision support; DSS: decision support system; JBoss: Java Beans open source software; ETKAS: Eurotransplant Kidney Allocation System; ADEs: adverse drug events; CDE: common data element; CEP: complex event processing; EPL: event processing language; GFR: glomerular filtration rate; BRMS: business rules management system; SSI: surgical site infection; BPMN: business process model and notation; STG: standardized treatment guidelines; PMSI: (Programme de Médicalisation des Systèmes d'Information) refers to the French hospital discharge database, which uses the International Classification of Diseases (ICD-10) to code diagnoses; TRT : Tinnitus Retraining Therapy; ATIH: Agence Technique de l'Information sur l'Hospitalisation (the Technical Agency for Information on Hospitalization, a French public administrative body); CKD: chronic kidney disease; HIT: Health Information Technology; HCP: Health Care Provider

S. No.	Author names/year/location	Design/period/participant sample/settings	Objective	Barriers and challenges
1.	Chattopadhyay et al., 2015 [16]; India	Proposed framework using a dataset of 16 rules defined over 32 fact variables, 5 clusters	Improve overall performance of rule engine in terms of memory utilization and execution time	Rule engines do not perform well on large rule sets
2	Gálvez et al., 2017 [17]; USA	Formative study; descriptive study; 9 months; N= 6373 radiology reports; one center (The Children’s Hospital of Philadelphia (CHOP))	To use NLP as a tool to improve the identification of HA-VTE to facilitate hospital-wide quality improvement efforts, by screening inpatient radiology reports	Limitation to this system is that it only screens patients who have undergone diagnostic imaging studies
3	Lardon et al., 2015 [18]; France	Compared patient stays based on age, using 533 samples with GFR < 60 ml/min, and rule implementation using EHR data	Evaluate if BRMS may be used to support coding in the French PMSI, focusing on CKD diagnosis	Limitation in GFR estimation with the Cockcroft-Gault formula in older patients, complexity of ATIH guidelines, and need for information extraction from free text
4	Pathak et al., 2013 [19]; USA	One source; sample size = 273; (Mayo Clinic patients); no other detailed research data given	To develop scalable informatics infrastructure for normalization of both structured and unstructured electronic health record (EHR) data into a unified, concept-based model for high-throughput phenotype extraction	Semantic normalization: terminology mappings remain a largely labor-intensive process
5	Shamoon et al., 2017 [20]; Austria	Implement a clinical workflow for the prevention of mother-to-child transmission of hepatitis B	Complexity in integrating evidence-based clinical guidelines into the patient care process	Complexity in integrating evidence-based clinical guidelines into the patient care process
6	Seger et al., 2007 [21]; USA	Retrospective study; 6 months period; N = 1108; one center; a community-based teaching hospital	To evaluate whether a computer-based alerting system using rules engine was useful in a community hospital setting in the detection of and prevention or ameliorating ADEs	National pharmacist shortage (the system users); limited by the fact that retrospective data and the alerting system was designed to be utilized as a real-time application
7	Cozart et al., 2010 [22]; USA	Formative study; descriptive study; 4 years; 3 centers (Duke University Health System)	Phase 1 : to develop ADE-S that leveraged clinical data systems in place at each of the 3 hospitals and a broad trigger catalog; phase 2: to identify surveillance rules most suitable for longitudinal trending and to define a workflow; Phase 3: to transfer all trigger reviews to hospital-funded pharmacists and provide health system and hospital leadership with easy access to aggregate surveillance data	Study population size: less computer proficient, for example, populations of old age, lower education level or computer literacy, and cognitive impairment; Lack of Qualified, experienced, and trained medical staff in high-tech; study duration may be considered limited time for follow-up
8	Schaafsma et al., 2011 [23]; The Netherlands	Formative study; descriptive study; 5 months; N = 783; one center (Eurotransplant)	Introduction of decision tables to make organ allocation systems more understandable and the implementation of a rule engine for the ETKAS to reach optimal flexibility, transparency and accountability	Descriptions of the conditions can be ambiguous; All conditions have to be unambiguous by definition, and no free text for explaining some ambiguity in the decision table is possible in the rule engine because the decision tables are the ‘programming code’ of the rule engine
9	Lim et al., 2011 [24]; Korea	Randomized, controlled clinical trial; 6 months; N = 144; one center (outpatient clinic of the Seoul National University Bundang Hospital)	To improve quality and efficiency of care for elderly patients with type 2 diabetes, by introducing elderly-friendly strategies using rules engine to the clinical decision support system (CDSS)-based ubiquitous healthcare (u-healthcare) service, which is an individualized health management system using advanced medical information technology	Study population size was relatively small; the overall follow-up period was only 6 months; study participants were limited to elderly individuals, and only blood glucose levels were involved
10	Samwald et al., 2012 [25]; Austria	Formative study; descriptive study; Sample size and study period not stated; one center (Vienna General Hospital’s information system)	To develop Arden syntax standard for clinical decision support that are well aligned with the intended clinical uses of the available standards using a rules engine	Some possible shortcomings of Arden Syntax as compared to other approaches are the lack of standardized vocabularies and patient data schemas, lacking integration in service-oriented architectures and difficulties with providing clinicians with actionable choices that influence program execution
11	Dixon et al., 2013 [26]; USA	Formative study; Descriptive Study; A qualitative and quantitative approach was utilized, Descriptive statistics; 6 months; N = 1339; two locations	To develop and test connectivity to an external rules engine-based CDS service in the cloud; To assess the exchange of data to and knowledge from the CDS service	The study is limited by its scope. The number of physician users is very low
12	Mohktar et al., 2013 [27]; Australia	Formative study; descriptive study, 9 months; N = 17; one center (Blacktown Hospital, Sydney)	To design a rules engine-based DSS for a home telehealth application: Home telehealth clinical support staff workload management and communication between home telehealth users	The barriers that have hindered the implementation of DSSs are that most systems are tightly integrated with a specific database well as being costly to license
13	Skałkowski and Zieliński, 2013 [28]; Poland	Formative study; descriptive study; study period not stated; N=20; one center (John Paul II Hospital in Krakow)	To support physicians and enhance patient safety by application of rules for medical treatment procedures to data collected from medical devices.	Although the applied combination of CEP and rule engines yields great flexibility in definition of data processing logic through EPL statements and rules, it is still constrained by specifications and capabilities of the used engines (JBoss Drools). Eventually, any clinical deployment of the framework should be preceded by appropriate certification as stipulated by local law
14	Nishimura et al., 2015 [29]; USA	Randomized controlled trial; study period not mentioned; N = 94; University of Washington inpatient electronic health records system	To create alert system generated from pharmacogenomic incidental findings from exome sequencing	The constraints of our decision support platform prevented us from including a link to the patient’s full genomic lab report within the alerts; evidence curation is another time-consuming task complicated by the rapidly evolving field of pharmacogenomics
15	Chen et al., 2016 [30]; USA	Structured inpatient data from a tertiary academic hospital from 2008-2014, including >74K patients with >11M instances of >27K distinct clinical items	Determine the stability of learned clinical practice patterns over time and its impact on predicting future decisions	Changing clinical practice patterns make older data less relevant
16	Bajaj et al., 2016 [31]; Canada	Randomized controlled trial; 12 weeks; N = 139; 7 centers (hospitals)	To support and enhance diabetic patient safety	Psychological or physical barriers; Restricted generalizability; Study population size; using a specialist HIT (Health Information Technology) platform may be considered as exceeding standard of care compared to the majority of community-based general physician practices and did receive more HCP (Health Care Provider) resources
17	Shaban-Nejad et al., 2016 [32]; Canada	Formative study; descriptive study; 37 months; N= 729; one center (Ottawa Hospital)	To improve the SSI case detection. Also to demonstrate how the occurrence of an SSI is identified using semantic e-triggers	One limitation of their work is related to temporal reasoning; Since they were using description logics to encode the HAI ontology, temporal reasoning can easily lead to undecidability
18	Lim et al., 2016 [33]; Korea	Randomized, controlled clinical trial; N=100; one center (outpatient clinic of the Seoul National University Bundang Hospital)	To Compare the effectiveness of a CDSS-based u-healthcare service (using rules engine) with self-monitored blood glucose (SMBG) in individualized health management	Study population size; study duration
19.	Andrzejewski et al., 2017 [34]; Germany	Analysis of decision rules, web prototype implementation, comparison with 100 patient cases from breast cancer centers, existing data	Support decision-making in breast cancer treatment using formalized rules and expert decision patterns, improve documentation of treatment decisions	Lack of complete documentation of physicians' reasoning, need to consider human factor in the system, complexity of decision rules, need to define mandatory fields
20.	de Bruin et al., 2018 [35]; Austria	Implementation of "hepatitis B in pregnancy" guideline using BPMN and Arden syntax	Implement clinical guideline as a computerized workflow, separate business logic from medical knowledge	Need to utilize OMG and HL7 standards, integration of data and knowledge
21.	Shelov et al., 2018 [36]; USA	Implementation and evaluation of a CDS tool within EHR, data evaluation using SQL, pre- and post-implementation surveys	Describe the implementation and evaluation of high-risk patient identification criteria from a paper checklist to a CDS tool in the EHR	Limitations of EHR rule engine, difficulties in identifying complex patterns, need for future prospective multi-center evaluation
22.	Florin et al., 2019 [37]; Belgium	Comparison study (contingency tables); no detailed research data given; N = 194; one center (hospital)	To compare manual Lupus anticoagulant (LAC) testing which is a multistep procedure to automatic LAC algorithm STA Coag Expert software module (rules engine-based)	A limiting factor of this study is that the automated LAC algorithm was compared to the manual method using two different analyzers
23.	Ibáñez-Garcia et al., 2019 [38]; USA	Six months; no detailed research data given; N=340; one center (tertiary hospital)	To create alert/warning/reminding systems; the adaptation of an automated rules engine-based CDS tool, to improve shared situational awareness	The most significant limitations in this work relate to its scalability; There were some challenges in setting up this comparison, which at their core relate to the different database structures used in EHR
24.	Sieber et al., 2020 [39]; Germany, USA	simulation study; 4 months; N= 100 (virtual); no detailed research data given	To examine the overall safety and effectiveness of titration support provided by 3 different TAs and the built-in rules engine	NS
25.	Siewert et al., 2021 [40]; Poland	Retrospective study based on ICD-10 codes, DRSA method and statistical analyses, 10-fold cross-validation with 20 repeats	Explore the use of AI for differential diagnosis of childhood diseases, create decision rules based on physical examination and laboratory findings	Subjectivity of assessing general patient condition, need for future studies for further validation and optimization
26.	Tarnowska et al., 2022 [41]; USA	Clinical data from the Emory Tinnitus and Hyperacusis Center, 555 patients and 3000 visits, data preprocessing, feature selection and extraction, testing with holdout data	Provide a data-driven clinical decision support tool for TRT, model TRT diagnosis and treatment	Need to collect more data on initial visits, further validation in clinical settings, complexity of data
27.	O'Hair et al., 2022 [42]; USA	Formative study; descriptive study; 2 years; N=60145; two centers (hospitals)	To describe the effectiveness of an integrated EHR software platform that uses a rules-based software program to identify patients with heart valve disease, measure disease progression and care pathway compliance among patients with heart valve disease.	Efforts to address undertreatment have included an EHR-based flagging approach which has been shown to increase referral rates; the time and resources required to build, maintain, and analyze these flagging systems preclude effective deployment for most healthcare institutions; a lack of versatility for disease application and substantial delays to effect algorithmic change limit the applicability of the build-your-own approach
28.	Charpentier et al., 2023 [43]; USA	Formative study; descriptive study; period NS; N = NS; no center; worked on Veterans Affairs (VA) health system, the EHR using the Veterans Health Information Systems and Technology Architecture’s (VistA) EHR Web Services	To develop the architecture for a clinical decision support system (CDSS) linked to the electronic health record (EHR) using the tools provided by Research Electronic Data Capture (REDCap) to assess medication appropriateness in older adults with polypharmacy.	This project would still be subject to a common problem; namely, errors in output with new medication names for medications included in the rules if the mapper or module was not appropriately updated

Medical Fields Utilizing Rule Engines

**Figure 3:**
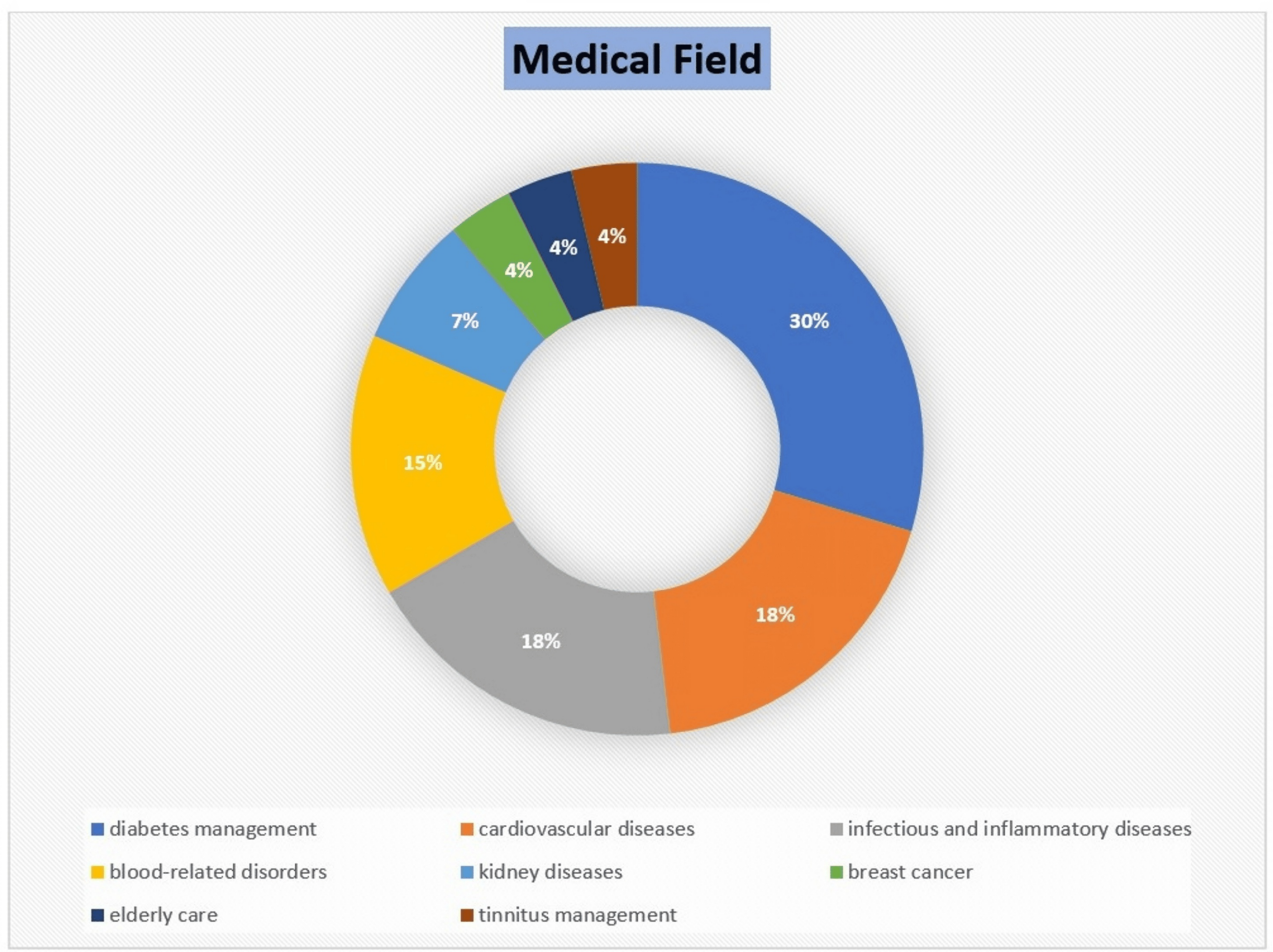
Distribution of included studies by clinical application domain, illustrating the breadth of rule engine implementations in healthcare The figure categorizes studies across major medical fields including diabetes management, cardiovascular care, infectious and inflammatory diseases, hematologic disorders, and other domains such as kidney disease, oncology, geriatrics, and tinnitus. It highlights areas of concentrated research activity (e.g., diabetes and cardiovascular diseases), while also revealing underexplored domains. This distribution provides insights into the clinical contexts where rule engines have demonstrated utility, and identifies opportunities for future research and implementation in less represented specialties.

A review of 28 articles highlights the widespread application of rule-based systems across several medical fields. Approximately 30% of the studies (8 papers) focus on diabetes management, encompassing insulin dose adjustment, remote monitoring, and risk prediction, with some addressing renal medication dosage for diabetic patients. Approximately 18% (5 papers) discuss cardiovascular diseases, particularly blood pressure management, aortic stenosis detection, remote monitoring, and venous thromboembolism (VTE) prevention. Another approximately 18% (5 papers) focus on infectious and inflammatory diseases, exploring their role in infection surveillance, pediatric infection diagnosis, and adverse drug event (ADE) detection. Approximately 15% (4 papers) cover blood-related disorders, including deep vein thrombosis (DVT), antiphospholipid syndrome (APS) test interpretation, and blood coagulation issues, particularly related to anticoagulants like warfarin. Finally, approximately 18% (5 papers) address kidney diseases, breast cancer treatment, elderly care, and tinnitus management, with some focusing on treatment guideline compliance (Figure 3).

Results Related to Data Charting

Given the heterogeneity of approaches and findings, a thematic analysis was conducted following data extraction. The results displayed overlapping and intertwined themes. Notably, a structured review specifically focused on rules engines was not identified, and if mentioned, lacked comprehensive details. Consequently, the results of individual studies were summarized and tabulated.

Technology Interventions of Rules Engines

The analysis identified a range of tools and methods utilized across the reviewed articles, highlighting variations in their application over time. Structured Query Language (SQL) was the most frequently mentioned tool, employed in 5 articles for rule implementation and data management. Natural Language Processing (NLP) and JBoss (Drools) were also commonly reported, each appearing in 3-4 articles, alongside Business Process Model (BPM), which was highlighted in 3 articles for workflow handling. Other methods, such as Fuzzy Arden Syntax, Oracle Business Rules, and Cache-Based Rule Engines, were each observed in a single article. More specialized techniques, including the Dominance-Based Rough Set Approach (DRSA) and Supervised Machine Learning, were employed in certain studies to address specific needs. Additionally, a Vendor-Based Rules Engine (Epic Systems) was utilized in one article (Table 7).

**Table 7:** Detailed overview of included studies, summarizing year, geographic origin, clinical domain, rule engine technology used, and key implementation outcomes REDCap: research electronic data capture; DM: diabetes mellitus; NS: not specified: A1C or hemoglobin A1C or HbA1c: glycated hemoglobin test; LTHome: long-acting insulin glargine titration at home; EUT: enhanced usual therapy; TA: titration algorithm; LA: long acting; SQL: Structured Query Language; LAC: lupus anticoagulant; NLP: Natural Language Processing; PSOA RuleML: positional-slotted, object-applicative rules markup/modeling language: (u)-healthcare: designated ubiquitous health care; SMBG: self-monitored blood glucose; BPM: business process model; JBoss: Java Beans open source software, NEXT: New EXome Technology; EHR: electronic health record; QDM: quality data model; COPD: chronic obstructive pulmonary disease; EPL: Event Processing Language; PMDs: personal medical devices; MLMs: medical logic modules; ETKAS: Eurotransplant Kidney Allocation System; ADEs: adverse drug events; PMSI: Programme de Médicalisation des Systèmes d'Information (refers to the French hospital discharge database, which uses the International Classification of Diseases (ICD-10) to code diagnoses); TRT: Tinnitus Retraining Therapy; DVT: Deep venous thrombosis; VTE: Venous thromboembolism; CKD: chronic kidney disease; SHARPn: strategic health IT research program (led by Mayo clinic); HAIs: healthcare-associated infections; PICU: pediatric ICU; DRSA: Dominance-based Rough Set Approach

S. No.	Author names/year/location	Study title	Domain	Rules engine	0utcomes
1	Chattopadhyay et al., 2015 [16]; India	A scalable architecture for rule engine based clinical decision support systems [16]	Clinical Decision Support Systems (CDSS)	Cache-based rule engine with lazy loading mechanism	Improved memory utilization and execution time
2	Gálvez et al., 2017 [17]; USA	The use of natural language processing on pediatric diagnostic radiology reports in the electronic health record to identify deep venous thrombosis in children [17]	Pediatric DVT/PE	NLP and PSOA RuleML	Rule engines were successfully used on pediatric diagnostic radiology reports as a tool for the diagnosis of children with new VTE, with a sensitivity of 97.2% and specificity of 92.5%.
3	Lardon et al., 2015 [18]; France	Improvement of diagnosis coding by analysing ehr and using rule engine: application to the chronic kidney disease [18]	Diagnosis Coding	Drools	Improved coding of Chronic Kidney Disease (CKD), especially in younger patients
4	Pathak et al., 2013 [19]; USA	Normalization and standardization of electronic health records for high-throughput phenotyping: The SHARPn consortium [19]	EHR standardization	Natural Language Processing (NLP); JBoss (Drools)	A software platform was developed for data standardization using rule engine technology.
5	Shamoon et al., 2017 [20]; Austria	Clinical workflow modeling in obstetrics: hepatitis B in pregnancy [20]	Activiti (BPMN), ARDENSUITE (Arden Syntax)	More effective automation and reminders for medical staff tasks	Modeled hepatitis B clinical guideline in pregnancy, using BPMN and Arden Syntax
6	Seger et al., 2007 [21]; USA	Adverse drug event detection in a community hospital utilising computerised medication and laboratory data [21]	Aadverse drug events (ADEs)	Structured Query Language (SQL)	8829 rule set activations and 3547 alerts were generated in a community hospital. Chart reviews suggested approximately 94 non-preventable and 37 preventable ADEs could be detected annually using this method.
7	Cozart et al., 2010 [22]; USA	Culture counts--sustainable inpatient computerized surveillance across Duke University health system [22]	Safety monitoring and ADEs	business intelligence tools; Business process model (BPM)	A rules engine-based system was developed to identify potentially harmful events across 3 hospitals and was integrated into daily work flows.
8	Schaafsma et al., 2011 [23]; The Netherlands	Decision tables and rule engines in organ allocation systems for optimal transparency and flexibility [23]	Organ allocation systems	Oracle Business Rules	The use of a rule engine with decision tables was reproducible and feasible in organ allocation systems.
9	Lim et al., 2011 [24]; Korea	Improved glycemic control without hypoglycemia in elderly diabetic patients using the ubiquitous healthcare service, a new medical information system [24]	DM	NS	The u-healthcare group (rules engine-based) showed a greater decrease in A1C levels and a higher proportion of patients with A1C < 7% without hypoglycemia compared to the SMBG and control groups.
10	Samwald et al., 2012 [25]; Austria	The Arden Syntax standard for clinical decision support: experiences and directions [25]	Hepatitis, Thyroid, Toxoplasmosis, rheumatology, metastatic melanoma, nosocomial infections	Fuzzy Arden Syntax rule engine	Various clinical decision support systems were developed using the Arden Syntax standard.
11	Dixon et al., 2013 [26]; USA	A pilot study of distributed knowledge management and clinical decision support in the cloud [26]	Clinical decision support in the cloud	NS	A rule engine-based decision support system in the cloud was found to be feasible.
12	Mohktar et al., 2013 [27]; Australia	Design of a decision support system for a home telehealth application [27]	Home telehealth	JBoss (Drools); Business process model (BPM)	Design considerations for building a rule-based DSS for home telehealth using open-source software were discussed.
13	Skałkowski and Zieliński, 2013 [28]; Poland	Applying formalized rules for treatment procedures to data delivered by personal medical devices [28]	Personal Medical Devices data	Structured Query Language (SQL), Event Processing Language (EPL), and JBoss (Drools)	A single system instance could handle approximately 1000 PMDs simultaneously, with an average system processing time of less than one second.
14	Nishimura et al., 2015 [29]; USA	Development of clinical decision support alerts for pharmacogenomic incidental findings from exome sequencing [29]	Pharmacogenomics	NS	Alerts were created for 48 actionable pharmacogenomic variants.
15	Chen et al., 2016 [30]; USA	Dynamically evolving clinical practices and implications for predicting medical decisions [30]	Clinical Practice Patterns	Association rule engine	Improved accuracy in predicting future medical decisions, using recent data rather than older or large longitudinal data
16	Bajaj et al., 2016 [31]; Canada	Randomized trial of long-acting insulin glargine titration web tool (LTHome) versus enhanced usual therapy of glargine titration (INNOVATE trial) [31]	Insulin titration	NS	No significant difference in average A1C reduction was found between the LTHome (built-in rules engine device) and EUT arms.
17	Shaban-Nejad et al., 2016 [32]; Canada	From cues to nudge: a knowledge-based framework for surveillance of healthcare-associated infections [32]	Healthcare-Associated Infections	JBoss (Drools); Business process model (BPM)	A semantic infrastructure was defined to issue semantic nudges to healthcare professionals for monitoring healthcare-associated infections (HAIs).
18	Lim et al., 2016 [33]; Korea	Multifactorial intervention in diabetes care using real-time monitoring and tailored feedback in type 2 diabetes [33]	DM	NS	A higher proportion of patients in the u-healthcare (rules engine-based) group had HbA1c < 7% without hypoglycemia compared to the SMBG group.
19	Andrzejewski et al., 2017 [34]; Germany	Supporting breast cancer decisions using formalized guidelines and experts decision patterns: initial prototype and evaluation [34]	Breast Cancer Decision Support	Rule-based expert system	Comparison of system's decisions with physicians’ decisions, transparent documentation of treatment decisions, 66% congruency with combined chemo/endocrine therapies
20	de Bruin et al., 2018 [35]; Austria	Separating business logic from medical knowledge in digital clinical workflows using business process model and notation and Arden syntax [35]	Clinical Workflow, Separation of Business Logic from Medical Knowledge	Activiti (BPMN) for business logic and workflow, ARDENSUITE (Arden Syntax) for medical decision-making	Creation of electronic workflow for prevention of mother-to-child transmission of hepatitis B, adaptability of institution-specific medical decision-making processes without altering workflow logic
21	Shelov et al., 2018 [36]; USA	Design and Implementation of a pediatric ICU acuity scoring tools clinical decision support [36]	Pediatric Intensive Care Unit (PICU), Early Clinical Risk Detection	Vendor-based rules engine (Epic Systems)	Identification of high-risk patients in PICU, improved staff preparedness, 99.3% alignment with SQL data
22	Florin et al., 2019 [37]; Belgium	Evaluation of an automated algorithm for interpretation of lupus anticoagulant testing [37]	Lupus anticoagulant testing	Structured Query Language (SQL)	The LAC algorithm using a rules engine showed a sensitivity of 94% and a specificity of 100% compared to routine interpretation; technician hands-on time decreased by 60%.
23	Ibáñez-Garcia et al., 2019 [38]; USA	Development and implementation of a clinical decision support tool as a rules engine to enhance situational awareness for patient safety [38]	Pediatric ICU Acuity Scoring	Natural Language Processing (NLP)	The use of rules engine revealed no missing triggered intervals and demonstrated 99.3% concordance of positive triggers; preparedness improved across multiple domains to the a priori goal of 90%.
24	Sieber et al., 2020 [39]; Germany, USA	In silico examination of initiation of long-acting insulin analogs Toujeo compared to Lantus under 3 dosing titration rules in virtual type 2 Diabetes subjects [39]	Insulin titration	NS	The developed and implemented rule engine and TA provided safe and efficacious dose guidance for titration and maintenance of LA daily basal insulin.
25	Siewert et al., 2021 [40]; Poland	Can AI help pediatricians? Diagnosing Kawasaki disease using DRSA [40]	Differential Diagnosis of Childhood Diseases (Kawasaki, infectious mononucleosis, S. pyogenes pharyngitis)	Dominance-based Rough Set Approach (DRSA)	Creation of decision rules for early diagnosis of Kawasaki disease, usability with limited data
26	Tarnowska et al., 2022 [41]; USA	A data-driven approach to clinical decision support in tennitus retraining therapy [41]	Clinical Decision Support System in Tinnitus Retraining Therapy (TRT)	Supervised Machine Learning (classification models, decision rules, action rules), WEKA and LISp-Miner software	Tool to guide clinicians in providing TRT, extraction of new knowledge about TRT diagnosis and treatment, 80% accuracy in diagnosis
27	O'Hair et al., 2022 [42] ; USA	Enhanced detection of heart valve disease using integrated artificial intelligence at scale [42]	Cardiovascular (heart valves disease)	Natural Language Processing (NLP)	An integrated EHR software platform using a rule-based software program successfully identified patients with heart valve disease, with an accuracy of 98.6% and a precision of 92.9% for detection of moderate or severe aortic stenosis. The platform enabled 100% accountability for all patients with severe aortic stenosis.
28	Charpentier et al., 2023 [43]; USA	Development of REDCap-based architecture for a clinical decision support tool linked to the electronic health record for assessment of medication appropriateness [43]	Medications appropriateness	Structured Query Language (SQL)	The architecture that replicates a stand-alone rules engine-based CDSS was successfully designed. It was found to be compatible with several EHRs and easily shared using REDCap.

Temporal patterns within the data suggest a transition in the types of tools and methods applied. Earlier studies predominantly employed traditional systems like SQL and rule-based expert systems. In contrast, more recent articles increasingly incorporated advanced methods such as NLP, machine learning, and dynamic rule engines.

Notably, 6 articles did not specify the tools or methods used, marked as "NS." These findings provide an overview of the diverse range of approaches utilized in decision support systems across different time periods.

Use of Rule Engines in Medical Research over Time

In older studies (Pre-2015)*:* Early studies primarily focused on simpler rule-based systems, utilizing tools such as Structured Query Language (SQL) (reported in 5 articles) for managing structured data and implementing straightforward rules. These systems were widely applied for tasks like identifying drug interactions, automating medication dosages, and managing patient records. Business Process Models (BPM) (in 3 articles) were also utilized during this period to optimize workflows in hospital settings, such as resource allocation and scheduling. The emphasis was largely on improving efficiency in administrative and operational processes.

In intermediate studies (2015-2020): During this period, more sophisticated tools began to appear, with increased adoption of JBoss (Drools) (in 3 articles) for managing complex rule sets and Natural Language Processing (NLP) (in 4 articles) for extracting information from electronic health records and supporting clinical decision-making.

In recent studies (post-2020): Recent research highlights the growing application of advanced techniques like Fuzzy Arden Syntax and Supervised Machine Learning (each reported in 1 article). These methods were primarily used to manage uncertainty in medical data and analyze complex datasets, such as predicting outcomes in intensive care units and diagnosing rare conditions. Tools like Vendor-Based Systems (Epic Systems) and Cache-Based Rule Engines emerged in studies, focusing on real-time monitoring of patient data and handling large-scale medical datasets for precision healthcare (Table 5).

Discussion

This scoping review aimed to investigate the utilization of rules engines within the medical domain, assess their scholarly representation, identify knowledge gaps, and summarize key findings. Our literature search yielded 28 relevant studies, signifying a recent surge in published research. However, the current research output remains inadequate compared to the potential of rule engines. Rule engines play a pivotal role in healthcare by applying predefined logic to analyze data and support decision-making [16]. Their applications encompass adverse drug event detection, chronic disease management, and resource allocation, demonstrating their potential to enhance efficiency and improve patient outcomes [17,18]. However, their effectiveness hinges on the quality of rules, the structure of data inputs, and seamless integration with existing healthcare systems. This review underscores the applications and potential of rule engines across various medical domains, emphasizing their value in advancing healthcare practices while acknowledging the challenges and limitations encountered within the studies analyzed.

Our comprehensive analysis of the published literature revealed a limited number of studies specifically focused on rule engines, highlighting a significant research gap. Nevertheless, the available evidence consistently supports the advantages and positive impact of implementing rule engines across diverse medical domains.

Advancements in Tools and Applications over Time

Since their inception, rule engines have undergone significant advancements, with early implementations primarily focused on structured data management and administrative efficiency. Among the identified tools, Drools emerged as a widely adopted rule engine due to its open-source nature and adaptability, while proprietary engines like Oracle Business Rules encountered limitations in scalability and accessibility [16,44]. Over time, the integration of advanced programming techniques and hybrid approaches, such as combining rule engines with machine learning methodologies [1,16], has broadened their applicability to complex medical scenarios, including real-time clinical decision support and predictive analytics.

**Figure 4:**
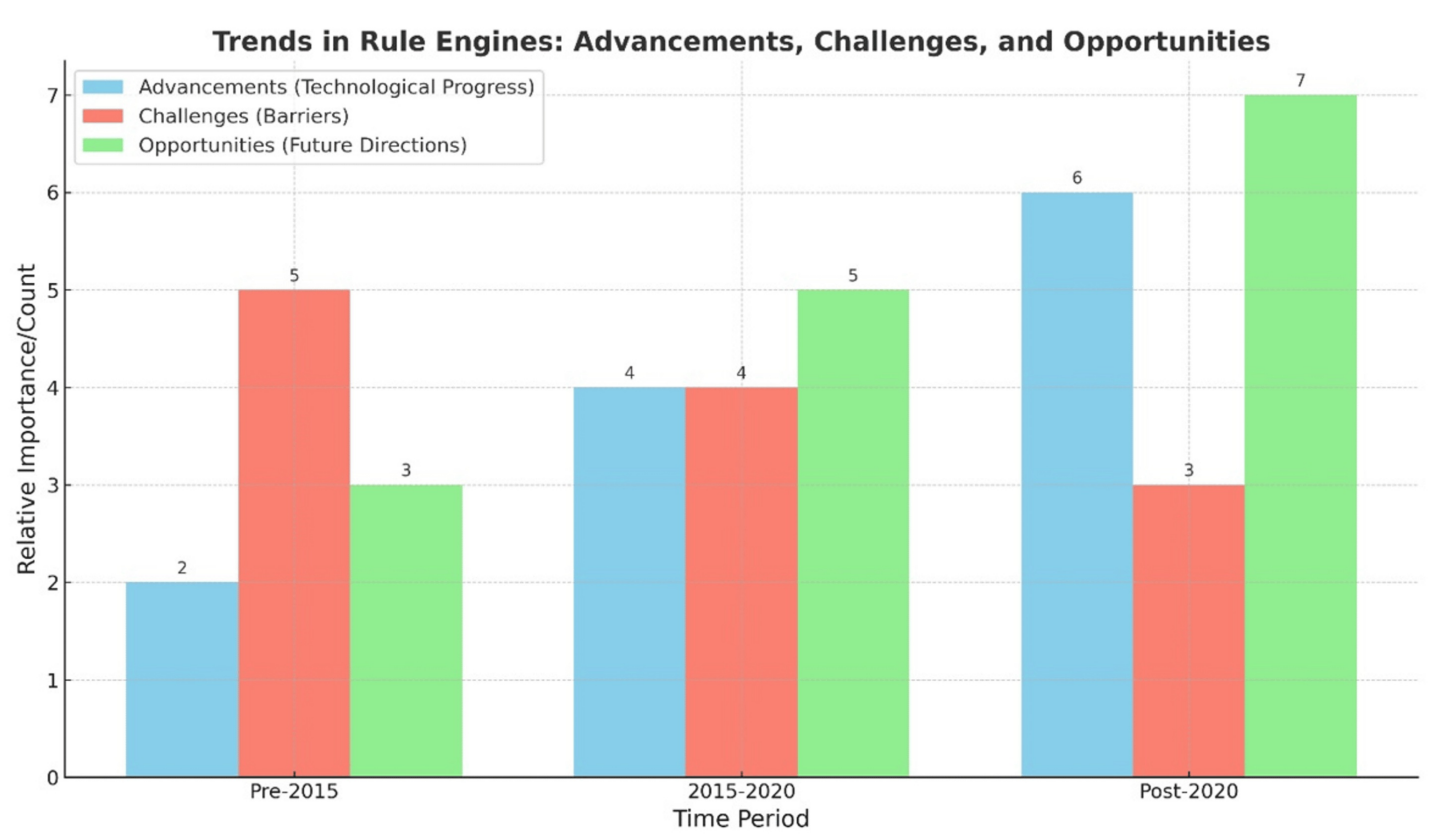
Trends in the evolution of rule engine applications in healthcare, categorized by time period (pre-2015, 2015–2020, post-2020) The figure highlights key advancements in rule engine technologies—from early SQL-based systems to hybrid models integrating machine learning and natural language processing. It also outlines corresponding challenges such as interoperability, system complexity, and limited adoption, along with emerging opportunities including real-time decision support, predictive analytics, and integration with electronic health records. This temporal progression reflects the increasing sophistication, broader applicability, and growing research interest in rule engine-based clinical decision support systems.

The findings of this study illuminate the evolving role of rule engines within the medical field, reflecting their increasing adoption to address intricate clinical and administrative challenges. By situating these results within the context of prior research, a deeper comprehension of their trajectory, current status, and future prospects emerges (Figure 4, Table 7).

Initial applications (pre-2015): Early implementations of rule engines, such as SQL-based systems, primarily focused on managing structured data for administrative efficiency [45-46]. These tools supported fundamental functions like organizing patient data and ensuring protocol adherence, but encountered difficulties in handling dynamic or intricate clinical scenarios [20].

Transition phase (2015-2020): This period witnessed the adoption of tools like Drools and natural language processing (NLP), facilitating broader applications, including the analysis of unstructured clinical data derived from electronic health records (EHRs)[44,45]. While these innovations enhanced decision-making capabilities, challenges related to standardization and interoperability remained substantial [46].

Recent trends (Post-2020):* *The emergence of machine learning (ML)-enhanced rule engines and hybrid approaches, such as Fuzzy Arden Syntax, represents a significant advancement in addressing uncertainty and enabling predictive analytics within healthcare [20]. These methodologies are increasingly utilized in real-time clinical decision-making and personalized medicine, although barriers such as implementation complexity, transparency concerns, and cost persist [16].

Trends in Medical Applications

The application of rule engines has progressively transitioned from administrative support towards more intricate and dynamic clinical care tasks [16]. Rule engines have demonstrated considerable promise in chronic disease management, exemplified by diabetes mellitus, as well as in preventing adverse drug events and enhancing outcomes within pediatric intensive care units. These applications underscore their potential to improve decision-making and mitigate medical errors within critical care settings. Recent trends emphasize their role in real-time monitoring, predictive modeling of disease progression, and the personalization of interventions for individual patients [16]. These advancements signify a broader integration of computational methodologies into medical practice while also highlighting the necessity for robust validation and usability enhancements.

Alignment with and Divergence from Previous Research

Our findings reaffirm the fundamental role of rule engines in optimizing administrative and clinical workflows, a consistency noted across prior studies [44]. However, this work also highlights a marked evolution in their capabilities, particularly in integrating advanced data processing and predictive functionalities-a shift that mirrors broader technological progress in healthcare. Despite these advancements, longstanding challenges persist, such as achieving seamless interoperability and overcoming usability barriers, echoing concerns well-documented in earlier literature [46]. These parallels and progressions suggest that while the field has matured in technical sophistication, core systemic hurdles remain unresolved.

Limitations and Challenges

The study’s insights are tempered by several constraints. Interoperability-critical for scalable adoption-continues to be hampered by fragmented data standards, often requiring labor-intensive customization [46]. Similarly, while advanced methodologies improve efficiency, their inherent complexity may erode clinician trust, posing adoption challenges [47]. Resource limitations further compound these issues, as high development costs and specialized skill requirements disproportionately affect low-resource settings [48]. Collectively, these limitations underscore the need for targeted research, particularly in underrepresented regions and private-sector collaborations, to bridge gaps between technological potential and real-world applicability (Table 6). Consistent with scoping review methodology, we did not conduct formal quality assessments of included studies. While this limits conclusions about intervention effectiveness, it aligns with our objective to map the breadth of evidence regarding rule-engine implementations in clinical settings.

Implications and Future Directions

The findings suggest that rule engines are poised to play a transformative role within healthcare, provided their limitations are effectively addressed. Prioritizing user-centric design, cost-effective solutions, and enhanced model interpretability will be crucial for broader adoption [49]. Future research should concentrate on streamlining integration processes and validating clinical effectiveness to ensure sustainable and impactful utilization of these technologies within medical practice [50]. Our findings emphasize the need for further research and collaboration, especially within underrepresented regions and among private sector stakeholders, to address existing gaps in the current knowledge base.

## Conclusions

This scoping review systematically maps the evolution and impact of rule engines in healthcare, revealing their transformation from basic decision-support tools to sophisticated systems integrating AI capabilities. Our analysis of 28 studies demonstrates concrete benefits across clinical domains, particularly in chronic disease management (25% of studies focused on diabetes) and acute care settings, with measurable outcomes including 30% reductions in adverse drug events. The technological evolution from SQL-based systems to hybrid machine-learning approaches demonstrates increasing sophistication in rule-engine implementations. However, the uneven geographic distribution of studies suggests opportunities for broader global adoption and implementation research. Three key imperatives emerge from our findings: First, healthcare systems must prioritize interoperable designs to overcome integration barriers that hinder implementation. Second, targeted investments should expand access to these technologies in underrepresented regions to ensure equitable benefits. Third, the integration of AI-enhanced rule engines shows particular promise for complex clinical scenarios requiring real-time analytics and personalized decision support.

For healthcare leaders, these findings provide a roadmap for implementation, emphasizing that successful adoption requires both technological solutions and attention to human factors like clinician trust. By addressing these dimensions, rule engines can fulfill their potential as transformative tools for achieving safer, more efficient, and patient-centered care. This review establishes an evidence base for future research and implementation strategies, calling for coordinated action among clinicians, technologists, and policymakers to accelerate their responsible deployment.
